# An Overview of Parameters Controlling the Decomposition and Degradation of Ti-Based *M_n_*_+1_*AX_n_* Phases

**DOI:** 10.3390/ma12030473

**Published:** 2019-02-04

**Authors:** It-Meng Low

**Affiliations:** Department of Applied Physics, Curtin University, GPO Box U1987, Perth, WA 6845, Australia; j.low@curtin.edu.au; Tel.: +61-8-9266-7544

**Keywords:** MAX phases, kinetics of decomposition, activation energy, vapor pressure, Avrami equation, Arrhenius equation, damage-tolerance

## Abstract

A critical overview of the various parameters, such as annealing atmospheres, pore microstructures, and pore sizes, that are critical in controlling the decomposition kinetics of Ti-based MAX phases is given in this paper. Ti-based MAX phases tend to decompose readily above 1400 °C during vacuum annealing to binary carbide (e.g., TiC*_x_*) or binary nitride (e.g., TiN*_x_*), primarily through the sublimation of *A* elements such as Al or Si, forming in a porous MX*_x_* surface layer. Arrhenius Avrami equations were used to determine the activation energy of phase decomposition and to model the kinetics of isothermal phase decomposition. Ironically, the understanding of phase decomposition via exfoliating or selective de-intercalation by chemical etching formed the catalyst for the sensational discovery of Mxenes in 2011. Other controlling parameters that also promote decomposition or degradation as reported in the literature are also briefly reviewed and these include effects of pressure and ion irradiations.

## 1. Introduction

MAX phases exhibit a unique combination of characteristics of both ceramics and metals and have unusual mechanical, electrical and thermal properties [1,2,3,4,5,6]. These materials are nano-layered ceramics with the general formula *M_n_*_+1_*AX_n_* (*n* = 1–3), where *M* is an early transition metal, *A* is a group *A* element, and *X* is either carbon and/or nitrogen. Similar to ceramics, they possess low density, low thermal expansion coefficient, high modulus and high strength, and good high-temperature oxidation resistance. Like metals, they are good electrical and thermal conductors, readily machinable, tolerant to damage, and resistant to thermal shock. The unique combination of these interesting properties enables these ceramics to be promising candidate materials for use in diverse fields which include automobile engine components, heating elements, rocket engine nozzles, aircraft brakes, racing car brake pads and low-density armor. In addition, MAX phases such as Ti_2_AlC and Ti_3_SiC_2_ have been shown to exhibit sufficient damage tolerance to irradiations which renders them as promising materials for high-temperature nuclear applications [7,8,9].

However, the high-temperature thermochemical stability in MAX phases has hitherto generated much controversy among researchers. For instance, several researchers have reported that Ti_3_SiC_2_ becomes unstable at temperatures greater than 1400 °C in an inert atmosphere (e.g., vacuum, argon, or nitrogen), by dissociating into Si, TiC*_x_* and/or Ti_5_Si_3_C*_x_* [10,11,12,13,14,15]. A similar phenomenon has been observed for Ti_3_AlC_2_ and Ti_2_AlN, whereby they decompose under vacuum to form TiC*_x_* and TiN*_x_*, respectively [16,17,18,19,20].

With regards to other studies, Zhang et al. [21] have reported Ti_3_SiC_2_ to be thermally stable up to 1300 °C in nitrogen but that above this temperature drastic degradation and damage occurs due to surface decomposition. Feng et al. [22] annealed the Ti_3_SiC_2_-based bulk samples at 1600 °C for 2 h and 2000 °C for 0.5 h under vacuum (10^−2^ Pa) and found that TiC*_x_* was the only phase remaining on the surface. According to Gao et al. [23] the propensity of the decomposition of Ti_3_SiC_2_ to TiC*_x_* is related to the vapor pressure of Si, i.e., the atmosphere to which the Ti_3_SiC_2_ is exposed. They believe that the partial pressure of Si plays an important role in maintaining the stability of Ti_3_SiC_2_ given that it has a high propensity to decompose in and N_2_, O_2_, or CO atmosphere at temperatures above 1400 °C. This process of surface-initiated phase decomposition has even been observed to commence as low as 1000–1200 °C in Ti_3_SiC_2_ thin films during vacuum annealing [24]. The large difference in observed decomposition temperatures between bulk and thin-film Ti_3_SiC_2_ has been attributed to the difference in diffusion length scales involved and measurement sensitivity employed in the respective studies. In addition, Ti_3_SiC_2_ has also been observed to react readily with molten Al, Cu, Ni, and cryolite (Na_3_AlF_6_) at high temperatures [25]. 

In contrast, Barsoum and co-workers [26] have shown that Ti_3_SiC_2_ is thermodynamically stable up to at least 1600 °C in vacuum for 24 h and in argon atmosphere for 4 h. They further argue that the reduced temperature at which Ti_3_SiC_2_ decomposes, as observed by others, is due to the presence of impurity phases (e.g., Fe or V) in the starting powders which interfere with the reaction synthesis of Ti_3_SiC_2_, and thus destabilize it following prolonged annealing in an inert environment [27]. However, mixed results have been reported by Radhakrishnan et al. [28]. In their investigation, Ti_3_SiC_2_ was shown to be stable in a tungsten-heated furnace for 10 h at 1600 °C and at 1800 °C in an argon atmosphere but dissociated to TiC*_x_* under the same conditions when a graphite heater was used. 

These conflicting results suggest that the thermochemical stability of MAX phases is still poorly understood, although its susceptibility to thermal decomposition is strongly influenced by factors such as purity of powders and sintered materials, temperature, vapor pressure, atmosphere, and the type of heating elements used. In addition, the nature of the microstructure of the decomposed surface layer formed during annealing remains controversial, especially in relation to the role of pore sizes in the decomposition kinetics at the near surface. 

In this paper, we provide a critical overview of the various parameters, such as annealing atmospheres, pore microstructure, and pore size, that are critical in controlling the decomposition kinetics of MAX phases. Other parameters that also cause decomposition or degradation as reported in the literature are briefly reviewed, and these include the effects of pressure and ion irradiations. 

## 2. Kinetics of Phase Decomposition

**Figure 1:**
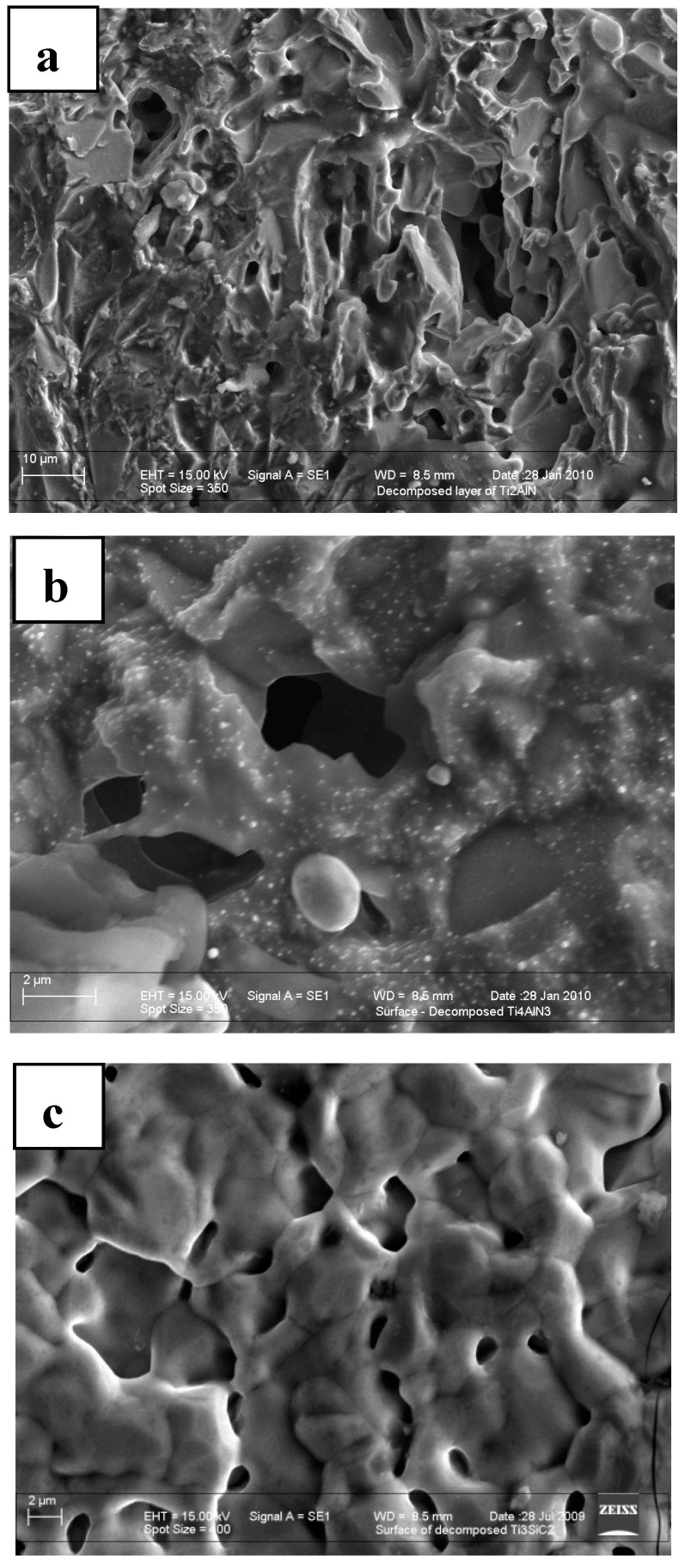

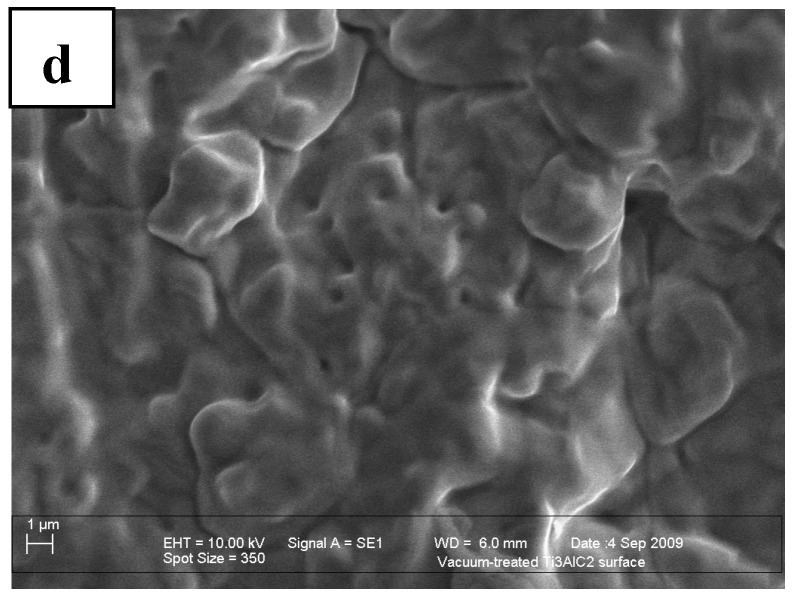
Scanning electron micrographs of the surface microstructures of vacuum-decomposed MAX phases; (**a**) Ti_2_AlN, (**b**) Ti_4_AlN_3_, (**c**) Ti_3_SiC_2_, and (**d**) Ti_3_AlC_2_ (bulk) Courtesy of AZom.com [10].

The activation energies calculated from the Arrhenius equation for the five MAX phases and the proposed reactions are summarized and listed in Table 1. All the calculated activation energies are positive except for that of bulk Ti_3_AlC_2_ [15]. However, when powder of Ti_3_AlC_2_ was used, a positive value of activation energy was obtained, which implies the importance of pore microstructures in the decomposition kinetics. A negative activation energy indicates that the rate of decomposition in Ti_3_AlC_2_ decreased with increasing temperature due to the formation of a dense TiC surface layer with very fine pores (<1.0 µm) which exert an increasing resistance to the sublimation process as the temperature increases (Figure 1d). In contrast, a more porous decomposed layer with coarser pores (>2.0 µm) formed in other MAX phases and in powdered Ti_3_AlC_2_ which enabled the sublimation of Al or Si to progress with minimum resistance, thus resulting in an increasing rate of decomposition with temperature (Figure 1a–c). In short, pore size plays a critical role in determining the value of activation energy and the rate of decomposition. Thus, the ability to manipulate the pore microstructure either through densification to reduce pore size or the engineering of pore free microstructure will allow the process of decomposition in MAX phases to be minimized or arrested.

The kinetics of isothermal phase decomposition as modelled using the Avrami equation and the Avrami exponents (*n*) of isothermal decomposition of the MAX phases are shown in Table 2. The low values (i.e., <1) of the exponent indicate that in all cases the decomposition is a highly restricted diffusion process, presumably of Al or Si from the bulk of the sample to its surface (from where it enters the vacuum of the furnace).

## 3. Controlling Parameters on Decomposition or Degradation

### 3.1. Role of Vacuum Annealing

**Figure 2:**
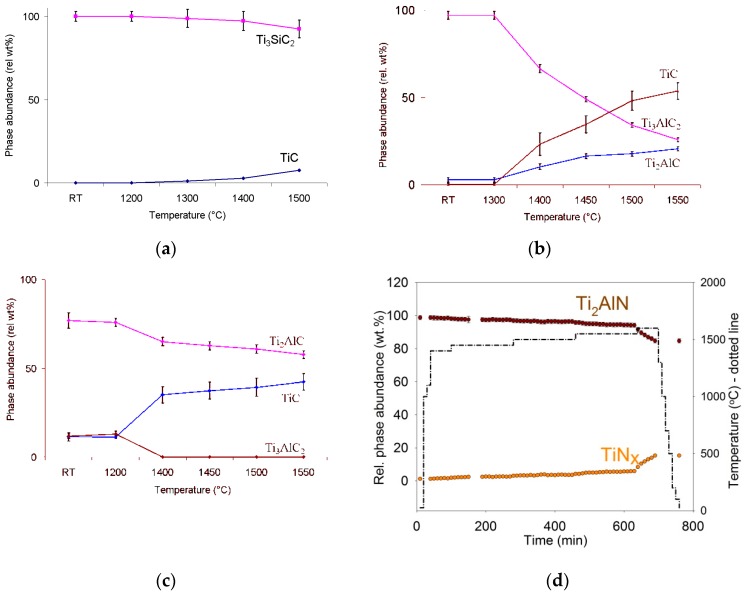
Phase abundance as a function of temperature for the decomposition of (**a**) Ti_3_SiC_2_, (**b**) Ti_3_AlC_2_, (**c**) Ti_2_AlC, and (**d**) Ti_2_AlN in vacuum. Courtesy of AZom.com [10].

The phase transitions in several *MAX* phases and their relative phase abundances under high vacuum (~10^−6^ torr) at various temperatures as revealed by in situ neutron diffraction are shown in Figure 2. A weight loss of ~4% was observed for decomposed Ti_3_SiC_2_, which may be attributed to the release of gaseous Ti and Si by sublimation during the decomposition process. For Ti_3_AlC_2_, its decomposition into TiC and Ti_2_AlC as a lower order MAX phase or intermediate phase was observed at ≥1400 °C. However, at higher temperatures, when compared to TiC, a smaller growth rate for Ti_2_AlC may indicate that Ti_2_AlC experienced further decomposition into TiC via the sublimation of Al, similar to decomposition of Ti_3_SiC_2_. In contrast to Ti_3_AlC_2_, no intermediate or lower order MAX phase was observed for the decomposition of Ti_3_SiC_2_. This difference can be attributed to the fact that Ti_3_SiC_2_ is the only stable ternary phase in the Ti-Si-C diagram. Figure 2d shows the excellent stability of Ti_2_AlN at 1500 °C for up to 350 min. 

In general, a weight loss of up to 20% that was observed as a result of decomposition for all the MAX phases can be attributed to the release of gaseous Al by sublimation during the decomposition process because the vapor pressures of the *A* elements exceed the ambient pressure of the furnace (i.e., ≤5 × 10^−5^ torr) at ≥1500 °C. Since the vapor pressure of a substance increases non-linearly with temperature according to the Clausius-Clapeyron relation [29], the volatility of *A* elements will increase with any incremental increase in temperature. 

Our work on vacuum-induced decomposition of MAX phases has been verified by Zeng and co-workers [30] who investigated the phase evolution of Ti_3_SiC_2_ in vacuum furnace up to 1500 °C. Decomposition of Ti_3_SiC_2_ via volatilization of Si was observed to commence at 1300 °C and became very severe at 1500 °C, resulting in a porous microstructure. An intermediate phase Ti_5_Si_3_C*_x_* was also observed to form between 1400 °C and 1450 °C. Similarly, Zhang et al. [31] have investigated the erosion of Ti_3_SiC_2_ due to the high energy intensity and high temperature of a vacuum arc. This material was found to be unstable under vacuum and decomposed at the sample surface, forming TiC*_x_*, with the concomitant ejection of Si vapour into the vacuum chamber.

The thermal stability of Ti_3_AlC_2_/Al_2_O_3_ composites in high vacuum was investigated by Chen and co-workers [32]. On the sample surface, Ti_3_AlC_2_ was observed to decompose with the formation of TiC_0.67_ with gaseous Al, followed by evaporation of Ti which resulted in the irregular morphology of non-stoichiometric TiC*_x_*. At the same time, the amount of Al_2_O_3_ particles decreased, with a prolonging of the dwell time. Finally, Al_2_O_3_ particles disappeared and a layer of non-stoichiometric TiC*_x_* forms on the sample surface. It is worth mentioning here that the nature of vacuum experienced by the samples (i.e., static or dyanamic) is likely to play the ultimate role in the decomposition of MAX phases. A static vacuum environment is perceived to be a closed system in which any vaporized elements are likely to recondense and react with the host sample at elevated temperature. On the other hand, an environment of dynamic vacuum has less chance of recondensation but has a greater driving force for the continual vaporisation of the “*A*” element, leading to rapid and extensive decomposition. In contrast to reported high thermal stability for bulk MAX-phase materials, their thin-film counterparts exhibited far inferior phase stability. For instance, Emmerlich and co-workers [33] synthesized Ti_3_SiC_2_ films using magnetron sputtering and these films were found to be stable during vacuum annealing at temperatures up to ~1000 °C for 25 h. However, annealing at 1100–1200 °C resulted in the rapid decomposition of Ti_3_SiC_2_ by Si out-diffusion along the basal planes via domain boundaries to the free surface with subsequent evaporation. Similarly, Ti_2_AlN thin films have been observed by Beckers and co-workers [34] to decompose as low as ~800 °C. The decomposition proceeded by outward Al diffusion and evaporation, followed by de-twinning of the as-formed Ti_2_N atomic layers into cubic TiN*_x_* and intermediate phases. The large difference in observed decomposition temperatures between bulk and thin-film Ti_3_SiC_2_ has been attributed to the difference in diffusion length scales involved and measurement sensitivity employed. These conflicting results suggest that the thermochemical stability of Ti_3_SiC_2_ and other MAX phases is still poorly understood, although their susceptibility to thermal decomposition is strongly influenced by factors such as vapour pressure, microstructure, furnace atmosphere and the nature of the vacuum (i.e., static or dynamic). 

In another study, Zhang and co-workers [35] investigated the phase stability of single-crystalline Ti_2_AlN thin film in ultra-high vacuum using in situ by X-ray photoelectron spectroscopy as a function of annealing temperature. The Ti_2_AlN thin-film was observed to be stable up to 600 °C but at 700 °C, Al was preferentially desorbed from the surface and became nearly undetected at 900 °C by XPS, and single-crystalline Ti_2_AlN with terrace morphology transformed into polycrystalline delta-TiN_1−*x*_ and xi-TiN_0.75−*y*_ phases with voids on the surface and reduced film thickness. The subsequent desorption of Al from the surface due to its high vapor pressure resulted in decreased Al composition, the void formation on the surface, and the decomposition of Ti_2_AlN.

Using a combined cathodic arc/sputter technique, Wang and co-workers [36] were able to fabricate dense and higher stability Ti_2_AlN coatings. Following vacuum annealing at 1000 °C for 1 h, these coatings were observed to remain intact with minimum degradation due to phase decomposition. This improved thermal stability is attributed to larger thickness and the denser structure, as well as the concomitant absence of defects (e.g., columnar grain boundaries, micro-cracks, and voids) which are the fast diffusion channels of Al to the surface under high vacuum conditions.

### 3.2. Role of Argon Atmosphere

As previous mentioned, Barsoum et al. [26] have shown that Ti_3_SiC_2_ is stable up to 1600 °C in argon for 4 h. However, mixed results have been reported by others [28], such as where Ti_3_SiC_2_ was shown to be stable in a tungsten-heated furnace for 10 h at 1600 °C in argon, but dissociated to TiC*_x_* when a graphite heater was used. In our previous work on decomposition of Ti_3_SiC_2_ at elevated temperatures in an argon atmosphere [14], we showed that this material remained quite stable at 1000 °C and that the phase concentrations of Ti_3_SiC_2_ and TiC impurity remained unchanged. However, at 1100 °C, Ti_3_SiC_2_ was observed to decompose and form additional TiC. The thermal dissociation process was slow below 1200 °C, but the process became quite rapid from 1250 to 1400 °C. A small amount of transient phase Ti_5_Si_3_C*_x_* was observed to form from 20 to 1400 °C. This phase is believed to form during the initial decomposition stage of Ti_3_SiC_2_ and converts to the stable TiC at elevated temperature. The existence of this transient phase at 1400–1450 °C has also been reported by Zeng et al. [30] in their study on phase decomposition of Ti_3_SiC_2_ under vacuum. It should be emphasized that an impurity such as TiC is unlikely to impart significant influence on phase decomposition because the process of decomposition involves primarily, if not exclusively, vaporization of the “*A* element”.

A similar phenomenon of MAX phase decomposition in argon has been reported by other investigators. The thermal stability of Cr_2_AlC in argon has been investigated by Xiao and co-workers [37]. According to these researchers, the produced Cr_2_AlC ceramic was stable up to 1500 °C in an Ar atmosphere, but decomposed into Al_8_Cr_5_ and Cr_23_C_6_ above 1500 °C. In another study on nanolaminated ternary boride Fe_2_AlB_2_ [38], the thermal stability of Fe_2_AlB_2_ at temperatures up to 1300 °C in Ar atmosphere was studied. It was observed that Fe_2_AlB_2_ intensively decomposed at 1236 °C in Ar atmosphere. In a separate study, Zhang and co-workers [39] investigated the thermal stability of bulk Zr_2_Al_4_C_5_ at elevated temperatures (20–2000 °C) under flowing argon. This material was observed to decompose initially at 1900 °C and became severely decomposed at 2000 °C, forming ZrC_1−*x*_ and Al_4_C_3_ on the surface. Similar to MAX phases, the mechanism of decomposition involved sublimation of Al and weaker covalent bonds between ZrC slabs and Al_4_C_3_ layers.

The thermal stability of Ti_3_AlC_2_ at 1250–1400 °C in a 10% CO-Ar atmosphere has been investigated by Gai et al. [40]. Decomposition of Ti_3_AlC_2_ was observed to commence at 1250 °C and a mixture layer of Ti(O,C) and Al_2_O_3_ formed at 1250–1300 °C. Thermal stability of MAX-phase coatings in argon atmosphere has also been investigated by several researchers. Using magnetron sputtering from Cr-Al-C composite targets at room temperature, Li and co-workers [41] prepared polycrystalline Cr_2_AlC coatings on a M38G superalloy. No phase decomposition was observed during subsequent annealing of these coatings in argon at 620 °C for up to 20 h. However, when annealing in argon was increased to 1100 °C, Smialek et al. [42] observed an interfacial reaction between Cr_2_AlC and an Ni-based LSHR alloy with the concomitant formation of an Al-depletion zone of Cr_7_C_3_. 

As with argon, the effect of hydrogen atmosphere on the high-temperature (1200–1400 °C) thermal stability of Ti_3_SiC_2_ was investigated by Chen and co-workers [43]. However, in this case the decomposed product was not TiC*_x_*. Instead, a dense TiSi_2_ layer formed on the sample surface at 1200 °C. The formation this TiSi_2_ layer caused the volume expansion and subsequently spalled off the surface at 1300–1400 °C. It remains a mystery why in a hydrogen atmosphere Ti_3_SiC_2_ decomposes to TiSi_2_ rather than the commonly observed TiC*_x_*.

### 3.3. Role of Pore Microstructure

**Figure 3:**
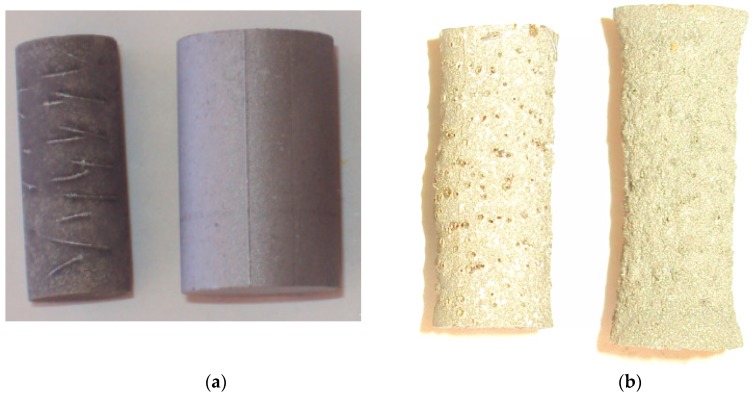
Surface conditions of Ti_4_AlN_3_ (left) and Ti_2_AlN (right) before (**a**) and after (**b**) thermal decomposition in vacuum.

It is well known that *A* elements such as Si and Al have high vapour pressure and become volatile at elevated temperatures. Thus, at the temperatures of well over 1500 °C used in this study, both Al and Si should become volatile and sublime readily and continuously in a dynamic environment of high vacuum. When the vapor pressure becomes sufficient to overcome ambient pressure in the vacuum furnace, bubbles will form inside the bulk of the substance and eventually appear as voids on the surface of the decomposed MAX phase. The evidence of surface void formation can be clearly discerned from the porous surface damage of decomposed Ti_2_AlN and Ti_4_AlN_3_ (Figure 3). These void formations are evident from the scanning electron micrographs of decomposed MAX phases shown in Figure 1. Si has a lower vapour pressure than Al, and this explains why Ti_3_SiC_2_ is more resistant to decomposition than Ti_3_AlC_2_ or Ti_2_AlN. In all cases, the kinetics of the decomposition process are driven mainly by a highly restricted out-diffusion and sublimation of high vapour pressure *A* element (e.g., Al and Si) from the bulk to the surface of the sample and into the vacuum, i.e.,

*M_n_*_+1_*AX_n_* → *M_n_*_+1_*X_n_* + *A* (1)

*M_n_*_+1_*X_n_* → (*n* + 1)*MX_n_*_/(*n*+1)_ (2)

As previously mentioned, research conducted in our laboratories shows that activation energies of phase decomposition are strongly influenced by the pore microstructures of decomposed material. A decomposed surface layer with very fine pores (<1.0 µm) has a ability to exert an increasing resistance to the sublimation process as the temperature increases. However, a more porous decomposed layer with coarser pores (>2.0 µm) would enable the sublimation of Al or Si to progress with minimum resistance, thus resulting in an increasing rate of decomposition with temperature. In short, pore size plays a critical role in controlling the kinetics of decomposition in MAX phases. Thus, the ability to manipulate pore microstructure either through densification to reduce pore size or engineering of pore free microstructures will allow the process of decomposition in MAX phases to be minimized or arrested.

### 3.4. Role of High Pressure

As mentioned above, a vacuum can provide a conducive environment for the vaporization of “*A* elements” (e.g., Si or Al) in MAX phases, leading to their subsequent decomposition. It would thus follow that the reverse (i.e., an increasing pressure) would provide hindrance to the escape of the “*A* elements” and hence improve the thermal stability at elevated temperature. However, this hypothesis has, surprisingly, been proven to be incorrect. For instance, the effect of high pressure (3–5 GPa) on the high-temperature phase stability of Ti_3_SiC_2_ has been investigated by Qin and He [44]. Similarly to high-vacuum annealing, which tends to destabilize MAX phases, the decomposition temperature of Ti_3_SiC_2_ decreases quickly with increasing pressure, and the low temperature limits of phase segregation of the sample Ti_3_SiC_2_ lie between 1100 °C and 1000 °C, 1000 °C and 900 °C, and 900 °C and 800 °C, under high pressures of 3, 4, and 5 GPa, respectively. The decomposed products observed are TiC, SiC, and TiSi*_x_*. The process of decomposition involved the outward diffusion of Si, followed by shrinking of material through relaxation of the Ti_3_C_2_ slabs, is similar to those observed in Ti_3_SiC_2_ thin film phase decomposition [24]. The released Si then reacts with Ti_3_C_2_ or TiC_0.67_ to form TiC, SiC, and TiSi*_x_* at high pressure-temperature conditions. Similarly, Ti_2_AlC is also unstable when the temperature is above 900 °C under 5 GPa pressure, and the decomposition temperature decreases with increasing pressure. The phase decomposition temperature of Ti_2_AlC detected was 890 ± 10 °C at 5 GPa and 1030 ± 10 °C at 4 GPa [45,46]. However, Ti_2_AlN has been observed to be more stable than Ti_2_AlC under similar high pressure-temperature conditions. An and co-workers [47] investigated the stability of Ti_2_AlN at 5 GPa and different temperatures of 700–1600 °C. Under a pressure of 5 GPa for 20 min, Ti_2_AlN was observed to maintain stability at high temperatures up to 1400 °C but decomposed into TiN and TiAl directly at 1500 °C. It should be noted that the effect of pressure on the thermal stability of MAX phases at elevated temperature has so far been exclusively investigated by only one group of researchers (i.e., He and co-workers) based in a Thai university. This important claim warrants further work and it needs to be independently verified by other researchers from various countries.

### 3.5. Role of Ion-Irradiation

Before MAX phases can be seriously considered for use by design engineers as a potential structural nuclear material for the fourth generation of reactors and future fusion reactors, their damage tolerance to irradiation requires careful investigations. So far, numerous studies have been conducted in this topic but mixed results have been obtained. An excellent work has recently been conducted by Tallman and co-workers [7,48,49] to investigate the effects of neutron irradiation on Ti_3_AlC_2_, Ti_2_AlC, Ti_3_SiC_2_, Ti_4_AlN_3_, and Ti_2_AlN. Following irradiation, these samples were observed to decompose to either TiC or TiN together with formation of defects such as dislocation loops, stacking faults, cavities, and even microcraking. Swelling and microcracking were also observed by Ang et al. [50,51] for Ti_3_AlC_2_–Ti_5_Al_2_C_3_ materials irradiated with neutrons, with subsequent reduction in their flexural strengths by up to 90%. Ion irradiation has also been observed to induce phase transition and decomposition of MAX phases. Qi and co-workers [52] irradiated Ti_3_SiC_2_ with 700 keV C ions over a range of fluences and sample temperatures. Irradiation at temperatures from room temperature to 270 °C resulted in decomposition of Ti_3_SiC_2_ to TiC. A similar phenomenon was observed for Ti_3_SiC_2_ when it was bombarded with MeV iodine [53], resulting in precipitation of TiC*_x_* due to surface decomposition.

### 3.6. Role of Miscellaneous Factors

This overview will not be comprehensive without mentioning other factors that also play a role in the degradation of MAX phases. In these materials, the *M_n_*_+1_*X_n_* layers are generally quite stable but the “*A*” layers are not, because the latter are weakly bound and susceptible to selective reaction with their environment by diffusing out of the basal planes. In general, the solubility of the *A* elements plays a deciding role in controlling the propensity of the reaction between max phases and various substances such as molten metals or salts as well as common bases and acids [7].

For instance, Bentzel et al. [54] investigated the interactions between SiC and pyrolytic carbon (PG) with Ti_2_AlC, Ti_3_AlC_2_, Ti_3_SiC_2_, and Cr_2_AlC. No reaction was observed between SiC and Ti_3_SiC_2_ or Cr_2_AlC after 30 h at 1300 °C. Additionally, no reaction was apparent between PG and Ti_3_SiC_2_. However, when heated under vacuum, the Ti_3_AlC_2_/SiC and Ti_3_AlC_2_/PG diffusion couples resulted in TiC layers that were ~50 μm and ~100 μm thick, respectively. Similarly, an interfacial layer (~10 μm) of Cr_3_C_2_ and Cr_7_C_3_ was seen to form on the Cr_2_AlC/PG diffusion couple. Chiker and co-workers investigated the infiltration behavior of Ti and Cu fillers into a Ti_2_AlC/Ti_3_AlC_2_ composite using a TIG-brazing process [55]. They observed that Ti_2_AlC/Ti_3_AlC_2_ decomposed into TiC*_x_* and Ti_3_AlC when it came into contact with molten Ti. The Ti_3_AlC_2_ phase decomposed completely in the presence of Cu or Ti filler-materials. On the other hand, Liu and co-workers [56] took advantage of partial decomposition in fabricating Ag/Ti_3_AlC_2_ composites with enhanced mechanical properties through the formation of Ag (Al) solid solution in the interfacial zone between Ag and Ti_3_AlC_2_. A similar concept was utilized by Septiadi et al. [57] in joining SiC_f_/SiC composites with thin tapes of Ti_3_AlC_2_ or Ti_3_SiC_2_. Again, strong joining was successfully accomplished through solid-state diffusion bonding courtesy of partial decomposition of the tapes. Lastly, the prized outcome of utilizing selective removal of the “*A*” element from MAX phases through chemical solution etching is the discovery of the sensational two-dimensional, ordered, double transition metals carbides, or Mxenes [58].

## 4. Concluding Remarks

The rediscovery of MAX phases by Barsoum and co-workers in the 1990s caused a sensation and research activities on these materials exploded in the late 1990s and early 2000s, particularly from researchers in China. However, this flurry of research activities, especially on synthesis and properties, has started to wane in recent years, partly due to the inability to find suitable applications and to commercialize them because of cost. The issue of thermal stability described above may have also played a part. Before MAX phases can be widely used in harsh and extreme environments such as aircraft engine components, rocket engine nozzles, or structural materials for Generation IV nuclear reactors, the issues pertaining to their susceptibility to thermal decomposition need to be fully addressed. 

Ironically, it is the understanding of phase decomposition via de-intercalation of these materials that has laid the foundation or catalyst for the eventual discovery of Mxenes. The glory days of MAX phases are now well and truly over and the excitement is now focused on Mxenes. Nevertheless, it is heartening to notice that very active research activities on MAX phases, especially on damage tolerance to irradiations, are still going on with a view to utilizing these materials for high temperature nuclear applications.

## Figures and Tables

**Table 1:** Comparison of the kinetics of decomposition in six MAX-phase samples. Courtesy of AZom.com [10].

MAX Phase	Activation Energy (kJ mol^−1^)	Pore Size (µm)	Proposed Reactions
Ti_3_SiC_2_	169.6	1.0–3.0	$Ti_{3}SiC_{2}\to 3TiC_{0.67}+Si_{\left(g\right)}$
Ti_3_AlC_2_ (bulk)	−71.9	0.5–0.8	$Ti_{3}AlC_{2}\to 3TiC_{0.67}+Al_{\left(g\right)}$
Ti_3_AlC_2_ (powder)	71.9	>1.0	$Ti_{3}AlC_{2}\to 3TiC_{0.67}+Al_{\left(g\right)}$
Ti_2_AlC	85.7	2.0–10.0	${\mathrm{Ti}}_{2}\mathrm{AlC}\to {2\mathrm{TiC}}_{0.5}+{\mathrm{Al}}_{\left(g\right)}$
Ti_2_AlN	573.8	2.0–8.0	${\mathrm{Ti}}_{2}\mathrm{AlN}\to {2\mathrm{TiN}}_{0.5}+{\mathrm{Al}}_{\left(g\right)}$
Ti_4_AlN_3_	410.8	1.8–3.0	Ti_4_AlN_3_ → 4TiN_0.75_ + Al_(g)_

**Table 2:** Comparison of the Avrami decomposition kinetics in MAX phases. Courtesy of AZom.com [10].

MAX Phase	Avrami Exponent (n)	Avrami Constant (k) (min^−n^)
Ti_4_AlN_3_	0.18	0.37
Ti_2_AlN	0.62	0.004
Ti_3_AlC_2_	0.0023	0.93
Ti_2_AlC	0.11	0.608
Ti_3_SiC_2_	8.93 × 10^−7^	2
